# β-Hydroxybutyrate-induced mitochondrial dysfunction and oxidative stress in BMSCs are ameliorated by mitophagy activation

**DOI:** 10.3389/fcell.2026.1926968

**Published:** 2026-08-27

**Authors:** Tao Tang, Jing Zhou, Xianbo Jia, Jiahao Shao, Meigui Wang, Siqi Xia, Shuai Chen, Wenqiang Sun, Jie Wang, Songjia Lai

**Affiliations:** 1 College of Animal Science and Technology, Sichuan Agricultural University, Chengdu, Sichuan, China; 2 Farm Animal Genetic Resources Exploration and Innovation Key Laboratory of Sichuan Province, Sichuan Agricultural University, Chengdu, Sichuan, China; 3 College of Animal Science and Technology, Southwest University, Chongqing, China; 4 Hong Feng Dairy Cow Breeding Co., Ltd., Mianyang, China

**Keywords:** ketone body metabolism, ketosis cow, mitochondrial function, ROS, skeletal muscle

## Abstract

Ketosis is a common metabolic disorder in periparturient dairy cows and is characterized by elevated circulating BHBA concentrations. Although the effects of ketosis on hepatic metabolism have been extensively studied, its impact on skeletal muscle remains poorly understood. This study investigated the effects of BHBA on bovine muscle satellite cells (BMSCs) and the role of mitochondrial quality control in BHBA-induced cellular injury. BHBA treatment significantly inhibited BMSC proliferation, promoted apoptosis, increased intracellular and mitochondrial ROS accumulation, reduced antioxidant enzyme activities, and impaired mitochondrial membrane potential in a dose-dependent manner. BHBA also disrupted mitochondrial ultrastructure, altered the expression of mitochondrial respiratory chain genes, promoted mitochondrial fission, and suppressed mitophagy. Similar effects were observed in C_2_C_12_ myoblasts, indicating that the detrimental effects of BHBA on myogenic cells are conserved across different cellular models. Notably, activation of mitophagy alleviated BHBA-induced oxidative stress, reduced ROS accumulation, improved antioxidant capacity, and enhanced ketone body metabolism, whereas inhibition of mitophagy exacerbated these alterations. These findings demonstrate that BHBA directly induces oxidative damage and mitochondrial dysfunction in myogenic cells. Impaired mitophagy contributes to the progression of cellular injury, whereas enhancement of mitochondrial quality control confers protection. This study provides new insights into the cellular mechanisms underlying skeletal muscle metabolic dysfunction during bovine ketosis and identifies mitophagy as a potential therapeutic target.

## 1 Introduction

Dairy cattle are important sources of milk and meat, and their nutritional and health status directly affects production efficiency and farm profitability (Fadul-Pacheco et al., 2017). With increasing demand for high-quality animal products, improving dairy-cattle productivity and meat quality has become increasingly important (Kostovska et al., 2024; Drachmann et al., 2024). Skeletal muscle plays a central role in growth and development, while bovine muscle satellite cells (BMSCs), the principal myogenic stem-cell population, support muscle growth, regeneration, and repair (Sousa-Victor et al., 2022; Yun et al., 2023; Zhu et al., 2022). The proliferation, differentiation, and survival of BMSCs are regulated not only by extracellular signals and growth factors but also by cellular metabolic status (Yue et al., 2025). Under stressful conditions, oxidative stress and mitochondrial dysfunction can impair BMSC proliferation and survival. Excessive production of reactive oxygen species (ROS) can cause macromolecular damage and trigger apoptosis, thereby compromising muscle development and regeneration (Peng et al., 2022).

β-Hydroxybutyrate (BHBA) is both a ketone-body energy substrate and a metabolic signaling molecule present in the blood and tissues of dairy cows (Tufarelli et al., 2024). During negative energy balance, particularly in early lactation, adipose triglycerides are mobilized and hydrolyzed to release non-esterified fatty acids (NEFAs) (Zhu Y. et al., 2025; Chen et al., 2025). He triglycerides are hydrolyzed by lipases to release free fatty acids (FFA), which then enter the liver and are converted into acetyl-CoA through β-oxidation. Inside the liver cells, two acetyl coenzyme A molecules combine to form acetoacetic acid. Subsequently, under conditions of hypoxia or increased energy demand, it is converted into BHBA. This process is mainly due to the adaptation of dairy cows to negative energy balance (NEB) during the excessive stage and the metabolic response to the excessive mobilization of NEFAs (Mcart et al., 2013; Adewuyi et al., 2005). In addition to serving as an energy substrate, BHBA regulates multiple metabolic and signaling pathways and has been associated with metabolic health, production performance, and stress adaptation in dairy cows (Yang W. et al., 2025; Mu et al., 2025; Missio et al., 2022; Deng et al., 2015). Mitophagy is a key mitochondrial quality-control mechanism that selectively removes damaged mitochondria and helps maintain cellular energy homeostasis (Wang et al., 2026; Ding et al., 2023; Zhao et al., 2026). Mitochondrial dysfunction is often associated with cellular oxidative stress, with both factors mutually influencing and collectively impacting cell survival and functionality (Baechler et al., 2019; Kujoth et al., 2005). In the context of BMSCs, elucidating the interplay between mitophagy and oxidative stress is essential for understanding their role in skeletal muscle cell metabolism. Moreover, while research surrounding the metabolism of BMSCs has gradually increased, there remains a lack of in-depth systematic investigations regarding the influence of BHBA on these biological processes.

PGC1α is a transcriptional coactivator that regulates energy metabolism, mitochondrial biogenesis, oxidative-stress responses, and inflammatory signaling. In addition to promoting fatty-acid oxidation and mitochondrial metabolism, PGC1α suppresses excessive inflammatory responses through interactions with multiple signaling pathways, thereby contributing to mitochondrial homeostasis and cellular adaptation under metabolic stress (Ozaki et al., 2023; Jäger et al., 2007; Zhang et al., 2026; Gai et al., 2026; Wang et al., 2025). FOXO1 is a forkhead transcription factor involved in cell-cycle regulation, apoptosis, and cellular stress responses (Song et al., 2025). In muscle, altered FOXO1 activity is associated with muscle atrophy, apoptosis, and changes in cell survival (Hah et al., 2022). PGC1α and FOXO1 may interact in the regulation of oxidative metabolism and stress adaptation, although the nature of this interaction is context dependent (Li et al., 2026; Halling and Pilegaard, 2020). Examining their expression in BHBA-treated BMSCs may therefore provide insight into the cellular response to ketone-body-associated stress. Accordingly, this study evaluated the effects of BHBA on BMSC proliferation, apoptosis, oxidative stress, mitochondrial function, and mitophagy and assessed associated changes in PGC1α and FOXO1 expression. C_2_C_12_ myoblasts were included as a complementary and well-characterized myogenic model to determine whether BHBA-induced mitochondrial dysfunction and impaired mitophagy represent conserved responses across different myogenic cell systems and to facilitate subsequent mechanistic investigations (Ozaki et al., 2023; Jäger et al., 2007; Zhang et al., 2026; Song et al., 2025; Hah et al., 2022; Li et al., 2026; Halling and Pilegaard, 2020).

The aim of this study was to characterize the effects of increasing BHBA concentrations on BMSC proliferation, apoptosis, redox homeostasis, mitochondrial function, and mitophagy and to determine whether pharmacological modulation of mitophagy alters BHBA-induced injury. The findings are intended to provide a cellular framework for future *in vivo* studies of skeletal-muscle dysfunction during bovine ketosis.

## 2 Materials and methods

### 2.1 Isolation and culture of BMSCs

Skeletal-muscle tissue was collected from the right hind limb of neonatal Chinese Holstein calves obtained from Mianyang Hongfeng Dairy Farming Company (Tang et al., 2026). Under sterile conditions, surrounding fascia was removed, and the tissue was washed twice with phosphate-buffered saline (PBS; Servicebio, Wuhan, China) containing 2% penicillin-streptomycin (Gibco, Thermo Fisher Scientific, United States). The tissue was minced into approximately 1–2 mm^3^ pieces and digested in Dulbecco’s modified Eagle medium/nutrient mixture F-12 (DMEM/F-12; Gibco, Thermo Fisher Scientific, United States) containing 0.1% collagenase type II (Solarbio, Beijing, China) at 37 °C for 20 min, with gentle agitation every 5 min. Digestion was terminated by adding fetal bovine serum (FBS; Gibco, Thermo Fisher Scientific, United States) to a final concentration of 10%. The cell suspension was filtered through a 70-μm cell strainer, and the filtrate was seeded into T25 culture flasks. Cells were maintained at 37 °C in a humidified atmosphere containing 5% CO_2_, and the complete medium was replaced every 2–3 days. Cells were passaged at 70%–80% confluence. Passage-2 cells were cryopreserved in liquid nitrogen using a controlled-rate freezing protocol and subsequently used for the experiments.

### 2.2 Cell culture, β-hydroxybutyric acid (BHBA) and sodium β-hydroxybutyrate (NAHB) treatment

BMSCs were used as the primary bovine myogenic cell model. C_2_C_12_ myoblasts were included as a complementary, well-established myogenic model to determine whether BHBA-induced changes observed in BMSCs were reproducible in another muscle-cell system. C_2_C_12_ cells (CBP60252; COBIER, Nanjing, China) and BMSCs were cultured in growth medium supplemented with 10% FBS at 37 °C in 5% CO_2_. At approximately 70% confluence, cells were treated with 0, 1.2, 2.4, or 4.8 mM BHBA (773861; Sigma-Aldrich, United States) for 12 h. For direct comparison between BHBA and sodium β-hydroxybutyrate (NaHB; 54,965; Sigma-Aldrich, United States), cells were treated with 2.4 mM of either compound for 12 h. Treatment concentrations were selected on the basis of previous studies and their relevance to circulating BHBA concentrations in dairy cows (Kraushaar et al., 2023; Mohsin et al., 2024; Zhao et al., 2023). A 12 h exposure duration was selected based on preliminary time-course experiments, which showed that BHBA induced measurable changes in BMSC viability and mitochondrial-related responses while maintaining adequate cellular integrity for subsequent analyses.

### 2.3 The 3-MA, CCCP, and H_2_O_2_ treatment of BMSCs

Methyladenine (3-MA, Selleck, S2767, United States) treatment, when the cell density reached approximately 70%, the cells were treated with DMEM/F-12 containing 5 mM 3-MA for 12 h (Wu et al., 2010). For Carbonyl cyanide 3-chlorophenylhydrazone (CCCP, Selleck, S6494, United States) treatment, when the cell density reached approximately 70%, the cells were treated with DMEM/F-12 containing 20 μM CCCP for 12 h (Ding et al., 2010). For H_2_O_2_ (Sigma, 7722-84-1, United States) treatment, when the cell density reached approximately 70%, the cells were treated with DMEM/F-12 containing 400 μM H_2_O_2_ for 12 h. When the cell density reaches 70%, it is combined with BHBA for treatment. Specifically, DMEM/F-12 culture medium containing 4.8 mM BHBA and 3-MA, CCCP or H_2_O_2_ is added and the cells are treated for 12 h. CCCP was prepared in dimethyl sulfoxide (DMSO), and a matched concentration of DMSO was included as the vehicle control in the corresponding experiments.

### 2.4 CCK-8 and EdU

Cell proliferation and viability were assessed using a Cell Counting Kit-8 (CCK-8; Zomanbio, Beijing, China) and a 5-ethynyl-2′-deoxyuridine (EdU) assay kit (Beyotime, Guangzhou, China). For the CCK-8 assay, cells at approximately 70% confluence were treated with BHBA for the indicated periods (0, 12, 24, or 48 h). Subsequently, 10 μL of CCK-8 reagent was added to each well, and the cells were incubated at 37 °C in 5% CO_2_ for 2 h. Absorbance at 450 nm was measured using a microplate reader (Thermo Fisher Scientific, United States). For the EdU assay, cells were incubated with 10 μM EdU for 2 h, fixed, and stained according to the manufacturer’s instructions. EdU- and Hoechst-positive nuclei in the same fields were imaged using an inverted fluorescence microscope (Olympus, Tokyo, Japan), and images were analyzed with Image-Pro Plus 6.0 (Media Cybernetics, Rockville, MD, United States).

### 2.5 Transmission electron microscopy

Following treatment, BMSCs and C2C12 cells were collected and fixed in 3% glutaraldehyde (Macklin, Shanghai, China), followed by postfixation in 1% osmium tetroxide (EMCN, Beijing, China). Samples were dehydrated, infiltrated, embedded, sectioned, and stained using standard procedures. Ultrathin sections mounted on copper grids were examined using a JEM-1400FLASH transmission electron microscope (JEOL, Japan).

### 2.6 Detection of mitochondrial superoxide, viable mitochondrial activity, JC-1 staining, and ROS measurement

Mitochondrial superoxide was assessed using a mitochondrial superoxide detection kit (S0061S; Beyotime, Guangzhou, China), and mitochondrial mass was evaluated using MitoTracker Green (C1048; Beyotime, Guangzhou, China). After treatment, cells were incubated with MitoSOX Red working solution at 37 °C for 40 min, followed by MitoTracker Green working solution for 30 min and Hoechst working solution for 15 min. Images were acquired using an IX73 inverted fluorescence microscope (Olympus, Tokyo, Japan). Mitochondrial membrane potential was measured using a JC-1 assay kit (M8650; Solarbio, Beijing, China). Cells were incubated with JC-1 working solution at 37 °C for 20 min in the dark, washed twice with JC-1 staining buffer, and counterstained with Hoechst for 15 min. Images of JC-1 aggregates, JC-1 monomers, and Hoechst staining were acquired immediately using identical exposure and acquisition settings across groups. Intracellular ROS levels were measured using a DCFH-DA-based detection kit (CA1410; Solarbio, Beijing, China) according to the manufacturer’s instructions. Cells were incubated with DCFH-DA working solution at 37 °C for 20 min in the dark, washed twice with prewarmed PBS, and counterstained with Hoechst for 15 min. Fluorescence images were acquired immediately using identical microscope settings for all groups.

To minimize staining-related artifacts, all experimental groups were processed in parallel using identical probe concentrations, incubation times, washing procedures, imaging order, exposure times, and acquisition settings. Staining was performed in the dark or under minimal ambient light, and images were acquired immediately after staining. Control cells underwent the same medium changes and staining procedures but received no BHBA or NaHB.

### 2.7 Total protein extraction and Western blotting

Total protein was extracted using a total protein extraction kit (BC3710; Solarbio, Beijing, China), and protein concentrations were determined using a total protein assay kit (BB-474172; Bestbio, Shanghai, China). Equal amounts of protein (30 μg per lane) were mixed with loading buffer, denatured, separated using a One-Step PAGE Preparation Kit (ORISCIENCE, Chengdu, China), and transferred to 0.22-μm PVDF membranes (Merck Millipore) at 400 mA for 60 min. Precooled transfer buffer and ice packs were used to limit heat generation. Membranes were blocked in TBST containing 5% nonfat dry milk for 2 h at room temperature and incubated overnight at 4 °C with the appropriate primary antibodies, followed by the corresponding horseradish peroxidase-conjugated secondary antibody for 1 h at room temperature. Primary-antibody information is provided in Supplementary Table S1. Immunoreactive bands were visualized using enhanced chemiluminescence reagent (HAKATA, Shanghai, China) and captured using a Touch Imager Pro system (e-BLOT Life Science, Shanghai, China). Band intensities were quantified using ImageJ and normalized to the corresponding β-actin band (Tang et al., 2025). β-Actin expression was not significantly altered by BHBA under the experimental conditions (Supplementary Figure S1).

### 2.8 Total RNA extraction and qPCR analysis

Total RNA was extracted using RNAiso Plus (9109; Takara, Beijing, China). RNA concentration and purity were assessed using a NanoDrop 2000 spectrophotometer (Thermo Fisher Scientific, Waltham, MA, United States), and only samples that met the quality criteria were used. First-strand cDNA was synthesized using the PrimeScript RT Reagent Kit (Takara, Beijing, China); small-RNA cDNA was synthesized using the SYBR PrimeScript miRNA Reverse Transcription Kit (Takara, Beijing, China). Quantitative PCR (qPCR) was performed with SYBR Green qPCR Master Mix (Takara, Beijing, China) on a CFX96 system (Bio-Rad, Hercules, CA, United States) in a total reaction volume of 10 μL. β-Actin was used as the reference gene, and relative expression was calculated using the 2^−ΔΔCT^ method. Primer sequences are provided in Supplementary Table S2.

### 2.9 Detection of CAT, SOD, GSH-Px/GPX activity and MDA, H_2_O_2_ content

Catalase (CAT), superoxide dismutase (SOD), and glutathione peroxidase (GSH-Px/GPX) activities, together with malondialdehyde (MDA) and H_2_O_2_ contents, were measured using commercial kits (Solarbio, Beijing, China). After treatment, cells were counted with a hemocytometer to standardize the amount of cellular material used for lysis. Lysates were prepared according to the manufacturers’ instructions. For the CAT assay, the reaction was initiated by adding the final reagent, the plate was mixed immediately, and absorbance at 240 nm was recorded at 5 and 60 s. All samples were processed manually by the same operator using an identical procedure. SOD activity was measured at 560 nm, GSH-Px/GPX activity at 412 nm, H_2_O_2_ content at 415 nm, and MDA content at 532 and 600 nm. Values were calculated according to the formulas supplied with the respective kits.

### 2.10 Statistical analysis

Statistical analyses were performed using GraphPad Prism 9.0. Data are presented as mean ± SEM. For comparisons among multiple groups, one-way ANOVA followed by Tukey’s multiple comparisons test was used. For experiments involving two independent variables, two-way ANOVA followed by Tukey’s multiple comparisons test was performed. A value of *P* < 0.05 was considered statistically significant.

## 3 Results

### 3.1 The effects of BHBA and NAHB on BMSCs

**FIGURE 1:**
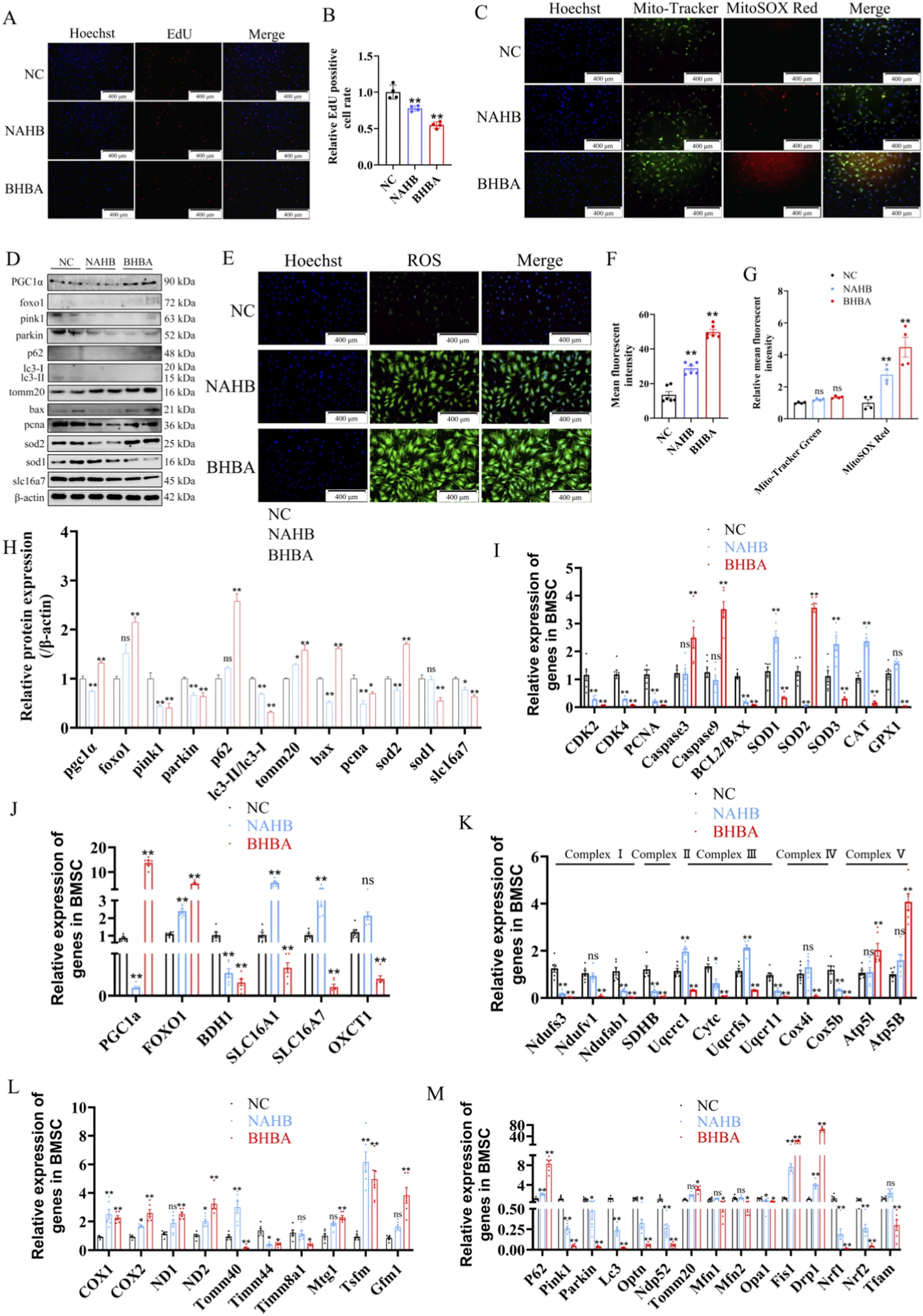
The Effects of BHBA and NAHB on BMSCs. **(A)** The picture of the EdU proliferation assay for BMSCs with BHBA and NAHB treatment. **(B)** The percent of EdU-positive cells (n = 3). **(C,G)** Assessment of mitochondrial ROS in BMSCs following treatment with BHBA and NAHB and statistical analysis of fluorescence intensity of mitochondrial ROS results using Image J (n = 3). **(D,H)** Relative selected protein; β-actin (PGC1α, FOXO1, Pink1, PARKIN, P62, LC3Ⅰ, LC3Ⅱ, TOMM20, BAX, PCNA, SOD2, SOD1, SLC16A7) protein levels were calculated by a grayscale scan. **(E,F)** Assessment of ROS in BMSCs following treatment with BHBA and NAHB and statistical analysis of fluorescence intensity of ROS results using Image J (n = 3). **(I)** The relative expression levels of genes related to cell proliferation, apoptosis, and oxidative stress in BMSCs treated with NAHB and BHBA (n = 6). **(J)** The relative expression levels of *PGC1α*, *FOXO1* and genes related to ketone body metabolism (n = 6). **(K)** The relative expression levels of genes related to mitochondrial complexes (n = 6). **(L)** The relative expression of mitochondrial-encoded genes, mitochondrial protein transport genes, and genes related to mitochondrial translation (n = 6). **(M)** The relative expression of genes related to mitochondrial autophagy, fusion, fission, and synthesis (n = 6). The data presented as means ± SEM. *P < 0.05; **P < 0.01.

To investigate the effects of BHBA on muscle tissue metabolism in dairy cows, we treated BMSCs with equal concentrations of BHBA (2.4 mM) and its precursor NAHB (2.4 mM). The concentration of 2.4 mM for BHBA and its precursor NAHB was selected based on physiologically relevant plasma levels in dairy cows and preliminary dose-response experiments, which demonstrated a clear metabolic response in muscle satellite cells (Yang W. et al., 2025). Detailed dose-response data are presented in Section 3.3. The results showed that both compounds significantly inhibited BMSCs proliferation (Figures 1A,B). BHBA treatment led to an increase in mitochondrial number and a significant accumulation of mitochondrial ROS. In contrast, NAHB had no noticeable effect on mitochondrial quantity but still significantly promoted mitochondrial ROS accumulation (Figures 1C,G). Furthermore, both treatments markedly increased total intracellular ROS levels (Figures 1E,F). The qPCR analysis revealed that BHBA suppressed cell proliferation, increased apoptosis, and reduced the expression of antioxidant genes in BMSCs. NAHB treatment also suppressed proliferation and increased apoptosis; however, it elevated the expression of antioxidant genes (Figure 1I). In addition, NAHB downregulated the expression of PGC1α and significantly upregulated genes involved in ketone body metabolism, again differing from the sequencing results (Figure 1J). BHBA treatment significantly downregulated the expression of mitochondrial complex I–IV genes and upregulated genes related to complex V, whereas NAHB had a milder effect (Figure 1K). Both BHBA and NAHB promoted mitochondrial transcription and translation, while inhibiting mitochondrial protein transport (Figure 1L). They also inhibited mitophagy, suppressed mitochondrial fusion and biogenesis, and promoted mitochondrial fission (Figure 1M). WB analysis showed similar trends (Figures 1D, H). Taken together, these results indicate that while BHBA and NAHB exert similar effects on BMSCs, the responses to BHBA are more consistent with the transcriptomic data and more closely reflect the metabolic changes observed in the muscle tissue of ketosis cows.

### 3.2 The effect of different concentrations of BHBA on BMSCs and C_2_C_12_

**FIGURE 2:**
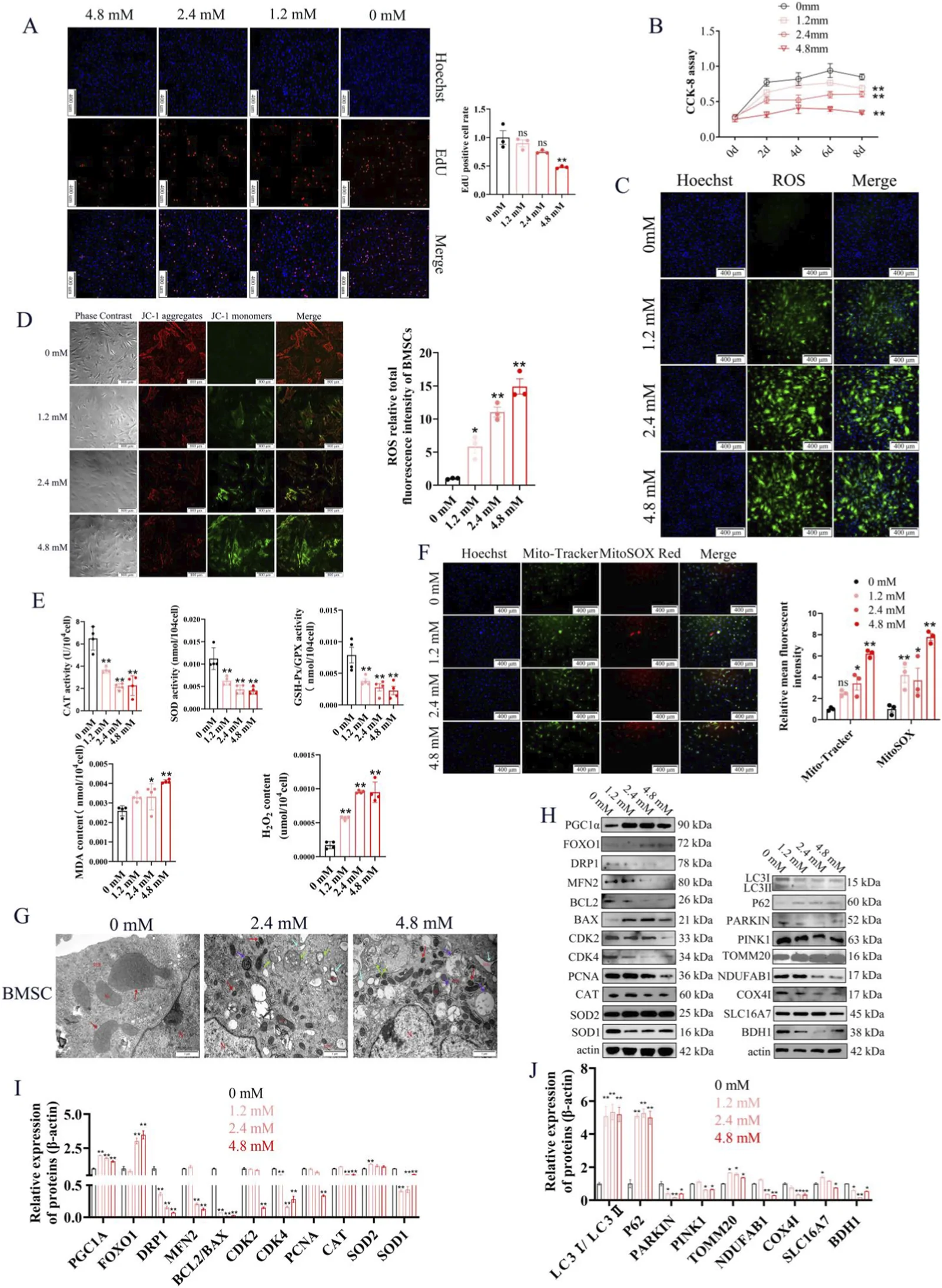
The effect of different concentrations of BHBA (0 mM, 1.2 mM, 2.4mM, 4.8 mM) on BMSCs. **(A)** The picture of the EdU proliferation assay for BMSCs with BHBA treatment for 12 h and the percent of EdU-positive cells (n = 3). **(B)** The 450 nm absorbance of BMSCs after treatment with BHBA (n = 6). **(C)** Assessment of ROS in BMSCs following treatment with different concentrations of BHBA and statistical analysis of fluorescence intensity of ROS results using ImageJ (n = 3). **(D)** JC-1 staining of mitochondrial membrane potential of different concentrations of BHBA (0 mM, 1.2 mM, 2.4mM, 4.8 mM) on BMSCs. **(E)** The detection of CAT, SOD, GSH-Px/GPX activity and the content of MDA and H_2_O_2_ in BMSCs after BHBA treatment (n = 4). **(F)** Assessment of mitochondrial ROS in BMSCs following treatment with different concentrations of BHBA and statistical analysis of fluorescence intensity of ROS results using ImageJ (n = 3). **(G)** Representative TEM micrographs of BMSCs with BHBA (0 mM, 2.4 mM, 4.8 mM) treatment. The red arrows represent mitochondria, the purple arrows represent autophagosomes, the blue arrows represent smooth endoplasmic reticulum expansion, the green arrows represent autophagosomes, RER refers to rough endoplasmic reticulum, SER refers to smooth endoplasmic reticulum, Mi refers to mitochondria, and N refers to the nucleus Scale bar = 1 μm. **(H–J)** Western blot analysis and detection of gray value (n = 3). Relative protein expression was normalized to β-actin. The data presented as means ± SEM. Statistical significance was analyzed using one-way ANOVA followed by Tukey’s multiple comparison test. *P < 0.05, **P < 0.0*P < 0.05; **P < 0.01.

In accordance with the actual production conditions of dairy cows, when the BHBA content in the cow’s body exceeds 1.2 mM, the cow is already in the clinical ketosis stage. Additionally, previous experimental results have shown that when the BHBA concentration exceeds 4.8 mM, there is a significant amount of BMSCs death. Therefore, this study used four BHBA concentrations (0 mM, 1.2 mM, 2.4 mM, 4.8 mM) to treat BMSCs for 12 h to investigate the effect of BHBA on bovine BMSCs. The results showed that BHBA significantly inhibited BMSCs proliferation in a dose-dependent manner (Figures 2A,B; Supplementary Figure S3B). A healthy and functional mitochondrial network is crucial for cellular quality control, while mitochondrial dysfunction is a key factor leading to the accumulation of intracellular ROS. Furthermore, the process of mitophagy is an essential biological process for clearing damaged mitochondria and maintaining normal mitochondrial function. BHBA induces the accumulation of mitochondrial ROS and intracellular ROS, as well as an increase in mitochondrial number (Figures 2C,F). The mitochondrial membrane potential decreases (Figure 2D), and the activities of CAT, SOD, and GSH-Px/GPX are reduced, while the levels of MDA and H_2_O_2_ increase (Figure 2E).

**FIGURE 3:**
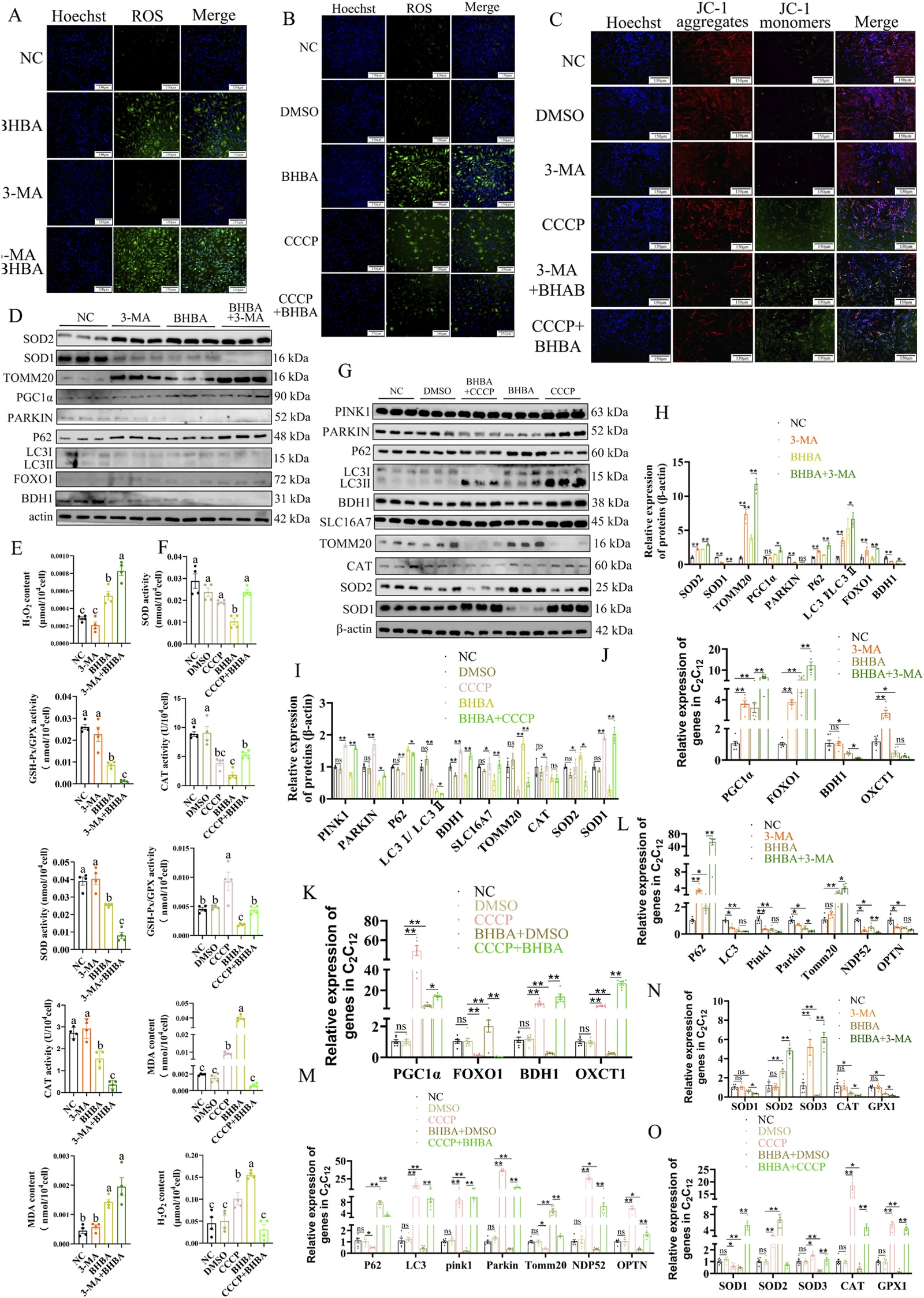
Enhancing the process of mitophagy might alleviate the damage caused by BHBA to C_2_C_12_. **(A,B)** Representative ROS images of Control, 3-MA (5 mM), CCCP (20 μM), BHBA (4.8 mM), BHBA+3-MA, and BHBA+CCCP groups. The CCCP powder is dissolved in DMSO to prepare a stock solution with a final concentration of 5 mM. **(C)** JC-1 staining of mitochondrial membrane potential of treatment with 3-MA, CCCP, and co-treatment with BHBA on C_2_C_12_. **(D,H)** Results of WB analysis after treatment with BHBA, 3-MA, and co-treatment of BHBA and 3-MA and detection of gray value (n = 3). **(E,F)** The detection of CAT, SOD, GSH-Px/GPX activity and the content of MDA and H_2_O_2_ in C_2_C_12_ (n = 4). **(G,I)** Results of WB analysis after treatment with BHBA, CCCP, and co-treatment of BHBA and CCCP and detection of gray value (n = 3). Relative protein expression was normalized to β-actin. **(J, K)** The relative expression levels of *PGC1α, FOXO1, BDH1* and *OXCT1* genes of CCCP and 3-MA treatment to C_2_C_12_ (n = 6). **(L,M)** The expression levels of mitochondria-autophagy related genes (n = 6). **(N,O)** The expression levels of antioxidant-related genes (n = 6). The data presented as means ± SEM. *P < 0.05; **P < 0.01.

To determine whether the effects of BHBA observed in primary bovine muscle satellite cells were conserved across different myogenic systems, C_2_C_12_ myoblasts were subjected to the same BHBA treatments. Similar to BMSCs, BHBA inhibited cell proliferation in a dose-dependent manner, promoted apoptosis, increased intracellular and mitochondrial ROS accumulation, reduced mitochondrial membrane potential, impaired antioxidant capacity, disrupted mitochondrial ultrastructure, suppressed mitophagy-related pathways, and altered the expression of mitochondrial dynamics- and respiratory chain-related genes (Supplementary Figure S2). These highly consistent phenotypic and molecular responses indicate that BHBA-induced mitochondrial dysfunction and oxidative stress are conserved features of myogenic cells rather than species- or cell type-specific phenomena. Therefore, C_2_C_12_ cells were subsequently used as a complementary mechanistic model to investigate the functional role of mitophagy in BHBA-induced cellular injury.

### 3.3 Enhancing the process of mitophagy might alleviate the oxidative damage caused by BHBA to C_2_C_12_

**FIGURE 4:**
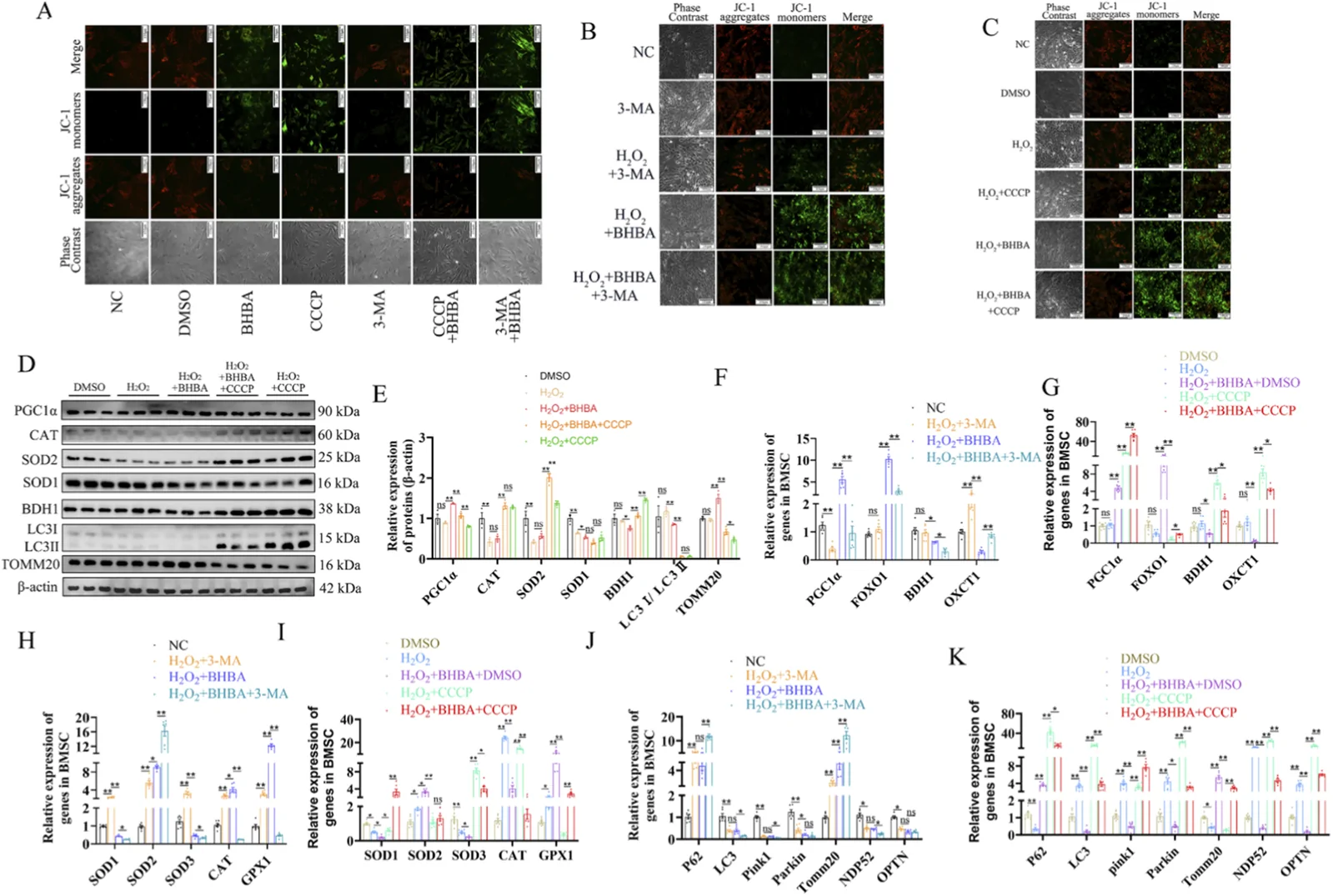
The increase of oxidative stress can exacerbate BHBA damage. **(A)** JC-1 staining of mitochondrial membrane potential of treatment with 3-MA, CCCP, and co-treatment with BHBA on BMSCs. **(B)** JC-1 staining of mitochondrial membrane potential of treatment with 3-MA, H_2_O_2_, and co-treatment with BHBA on C_2_C_12_. **(C)** JC-1 staining of mitochondrial membrane potential of treatment with CCCP, H_2_O_2_, and co-treatment with BHBA on C_2_C_12_. **(D,E)** Results of WB analysis after treatment with H_2_O_2_, CCCP, and co-treatment of BHBA and CCCP and detection of gray value (n = 3). **(F,G)** The relative expression levels of *PGC1α, FOXO1*, *BDH1* and *OXCT1* genes of H_2_O_2,_ H_2_O_2_+3-MA, H_2_O_2_+CCCP, H_2_O_2_+BHBA, H_2_O_2_+BHBA+3-MA, H_2_O_2_+BHBA + CCCP and H_2_O_2_+BHBA + DMSO treatment to C_2_C_12_ (n = 6). **(H,I)** The expression levels of antioxidant-related genes (n = 6). **(J,K)** The expression levels of mitochondria-autophagy related genes (n = 6). The data presented as means ± SEM. Two-way ANOVA followed by Tukey’s multiple comparison test. *P < 0.05; **P < 0.01.

The above results indicate that after treatment of BMSCs and C_2_C_12_ with BHBA, significant mitochondrial damage occurs within the cells. The structure and function of the mitochondria are impaired, leading to the production and accumulation of mitochondrial ROS, which ultimately results in cellular oxidative damage. Mitophagy can effectively remove damaged mitochondria within cells, thereby maintaining normal mitochondrial function. To further determine the role of mitophagy, C_2_C_12_ cells were treated with the mitophagy inhibitor (3-MA) alone, the mitophagy activator (CCCP) alone, or in combination with BHBA. The results showed that inhibition of mitophagy increased ROS levels after BHBA treatment (Figure 3A), whereas activation of mitophagy alleviated intracellular ROS levels induced by BHBA (Figure 3B), with little effect on mitochondrial membrane potential (Figures 3C, 4A). In addition, after inhibiting mitophagy, compared to BHBA treatment, intracellular CAT, SOD, GSH-Px/GPX activity further decreased, and MDA and H_2_O_2_ levels further increased, while CCCP treatment showed the opposite trend (Figures 3E,F). QPCR results showed that 3-MA successfully inhibited the mitophagy process in cells, and CCCP promoted the process (Figures 3L,M). Moreover, 3-MA significantly increased the expression of *PGC1α* and *FOXO1* genes, whereas CCCP suppressed the expression of FOXO1. In the ketone body metabolism process, compared to the BHBA group, 3-MA further inhibited the expression of BDH1 and OXCT1 genes, while promoting mitophagy, resulting in increased ketone body metabolism (Figures 3J,K). Similarly, after inhibiting mitophagy and BHBA treatment, oxidative stress levels further increased, whereas promoting mitophagy improved this process. However, either promoting or inhibiting mitophagy alone also caused an imbalance in intracellular oxidative stress levels (Figures 3N,O). To verify the accuracy of the qPCR results, the study also conducted WB experiments, which were consistent with the quantitative results (Figures 3D and G-I). The study also found that increased oxidative stress levels further disrupted mitochondrial membrane potential, decreased mitophagy, and increased oxidative damage, while increasing CCCP alleviated this process (Figure 4). These results suggest that the mitophagy process can alleviate oxidative damage caused by BHBA, and enhancing mitophagy can improve mitochondrial function and increase KBs metabolism.

## 4 Discussion

Ketosis is a prevalent metabolic disorder in periparturient high-yielding dairy cows and is characterized by excessive accumulation of ketone bodies, among which BHBA is considered the most stable and biologically relevant biomarker (Foster, 1988). Previous studies have focused primarily on hepatic lipid metabolism, immune dysfunction, mammary-gland performance, and reproductive impairment, whereas the effects of elevated ketone-body concentrations on skeletal muscle remain less well defined (Churchward-Venne, 2025; Bellato et al., 2023; Zhao et al., 2025). Skeletal muscle is a major peripheral tissue involved in energy expenditure, substrate utilization, and systemic metabolic homeostasis (Jun et al., 2024). Impaired satellite-cell function during ketosis could therefore compromise muscle regeneration and tissue maintenance. In the present study, BHBA reduced BMSC proliferation, increased apoptosis-related markers, induced oxidative stress, altered mitochondrial dynamics, and suppressed mitophagy. Pharmacological modulation of mitophagy further altered the severity of BHBA-induced oxidative injury, supporting an association between mitochondrial quality control and cellular tolerance to ketone-body-associated stress.

At 2.4 mM, BHBA produced more pronounced effects on BMSCs than NaHB. Both compounds reduced proliferation and increased intracellular ROS, but BHBA induced larger changes in mitochondrial mass, mitochondrial ROS, antioxidant-gene expression, and respiratory-chain-related transcripts. NaHB produced a more modest mitochondrial phenotype and, for several genes, a different transcriptional pattern. These observations indicate that BHBA and NaHB elicited distinct responses under the conditions examined. However, because NaHB was tested at only one concentration, the study cannot establish comparative dose-response relationships or identify the mechanisms responsible for these differences. Accordingly, the present data should not be interpreted as demonstrating that BHBA is intrinsically more harmful than NaHB across concentrations. Further studies incorporating multiple NaHB concentrations, matched physicochemical controls, and direct analyses of mitochondrial metabolism are needed.

The dose-response experiments provide evidence that BHBA disrupts BMSC viability and mitochondrial homeostasis. Increasing BHBA concentrations progressively reduced proliferation and altered apoptosis-related gene expression, indicating that muscle satellite cells are sensitive to excessive ketone-body exposure (Ren et al., 2025). This observation is particularly important given the indispensable role of muscle satellite cells in skeletal muscle maintenance, regeneration, and adaptive remodeling. Under physiological conditions, ketone bodies can serve as alternative oxidative substrates that contribute to cellular energy homeostasis. However, excessive BHBA accumulation may drive a transition from adaptive metabolic utilization to metabolic toxicity, thereby compromising cellular function and disrupting tissue homeostasis (Dumont et al., 2015; Tsuruta et al., 2024; Hay et al., 2026). The concentration-dependent reductions in CAT, SOD, and GSH-Px/GPX activities, together with increased MDA and H_2_O_2_ contents, indicate loss of redox homeostasis. The concurrent increases in total and mitochondrial ROS further suggest that mitochondria are both targets and potential amplifiers of BHBA-induced oxidative stress (Shirian et al., 2024).

Mitochondrial dysfunction may represent a critical mechanistic link between BHBA exposure and the compromised survival of BMSCs. In this study, BHBA treatment decreased mitochondrial membrane potential, altered mitochondrial ultrastructure, increased mitochondrial electron density, promoted mitochondrial condensation, and induced the accumulation of autophagosomes and autolysosome-like structures. These morphological and functional alterations indicate that BHBA imposes substantial stress on mitochondria. At the molecular level, BHBA downregulated the expression of genes encoding mitochondrial complexes I–IV while upregulating complex V–associated genes, suggesting disruption of electron transport chain integrity and impaired coordination of oxidative phosphorylation (Genserová et al., 2024). Inhibition of complexes I–IV may reduce electron transfer efficiency and increase electron leakage, thereby promoting mitochondrial ROS generation. Concurrently, the upregulation of genes associated with complex V may represent a compensatory cellular response to maintain ATP synthesis under conditions of respiratory chain impairment (Okoye et al., 2023). However, when upstream electron transport is impaired, such compensatory responses are unlikely to fully restore mitochondrial function. This may explain the persistent accumulation of ROS and the decline in mitochondrial membrane potential observed following BHBA treatment.

BHBA also increased mitochondrial mass and PGC1α expression despite evidence of mitochondrial injury (Chi et al., 2023). Increased PGC1α expression may reflect an adaptive response to mitochondrial stress (Killackey et al., 2022); however, this interpretation remains speculative because mitochondrial biogenesis and respiratory function were not directly assessed. MitoTracker Green fluorescence indicates mitochondrial mass but does not distinguish increased biogenesis from impaired mitochondrial turnover or organelle accumulation. Similarly, changes in mitochondrial transcription- and translation-related genes do not establish functional mitochondrial biogenesis. Future studies should measure mitochondrial DNA copy number, oxygen-consumption rate, ATP production, and mitochondrial biogenesis and turnover to define the biological significance of PGC1α upregulation. While cells may increase mitochondrial biogenesis or accumulate more organelles under these conditions, the resulting mitochondria are structurally aberrant and functionally impaired, and they cannot be efficiently removed by mitochondrial quality control pathways. The persistence of dysfunctional mitochondria further promotes ROS generation and aggravates oxidative stress–mediated cellular damage (He et al., 2021).

Mitochondrial fusion and fission are essential for maintaining mitochondrial architecture, metabolic adaptability, and quality control. Fusion allows exchange of mitochondrial contents, whereas fission can segregate damaged mitochondrial components for subsequent elimination by mitophagy (Ma et al., 2023). I In this study, BHBA altered the expression of genes and proteins involved in mitochondrial dynamics and was associated with mitochondrial fragmentation and impaired mitophagy. When mitophagy is insufficient, fragmented and dysfunctional mitochondria may accumulate and contribute to ROS production, membrane depolarization, and apoptotic signaling (Guhathakurta et al., 2023; Pham et al., 2024). Nevertheless, the present data primarily demonstrate associations among these processes; targeted genetic manipulation and direct mitophagy-flux measurements are needed to establish their temporal and causal relationships.

Changes in PGC1α and FOXO1 expression were associated with BHBA-induced cellular stress in both BMSCs and C2C12 cells. PGC1α regulates mitochondrial metabolism and oxidative-stress responses (Halling and Pilegaard, 2020; Dumesic et al., 2025; Praharaj et al., 2024; Abu Shelbayeh et al., 2023), whereas FOXO1 participates in cell-cycle control, apoptosis, and stress adaptation (Yang Y. et al., 2025; Zhang et al., 2021). In the present study, FOXO1 expression was also markedly altered following BHBA treatment, particularly in association with suppressed cell proliferation and enhanced apoptosis. The upregulation of FOXO1 may represent a protective cellular response to oxidative stress and injury, aimed at restoring cellular homeostasis through the transcriptional regulation of antioxidant defense pathways. Specifically, FOXO1 has been shown to induce the expression of a variety of antioxidant enzymes, thereby facilitating the removal of excess ROS and mitigating oxidative damage. However, the elevated FOXO1 expression observed following BHBA exposure may also reflect a cellular stress-sensing response to excessive BHBA accumulation and ROS generation.

The use of C_2_C_12_ cells provided complementary evidence that BHBA produces similar alterations in two myogenic cell models. Nevertheless, BHBA is not universally cytotoxic. Previous studies have reported beneficial metabolic effects of BHBA under physiological conditions such as fasting or caloric restriction (Parker et al., 2018; Benjamin et al., 2022). The divergent findings across experimental contexts suggest that the biological effects of BHBA depend on concentration, exposure duration, cell type, and metabolic state. Species-specific and developmental differences between bovine primary BMSCs and murine C_2_C_12_ myoblasts must also be considered. BMSCs more closely represent bovine skeletal muscle, whereas C_2_C_12_ cells offer a reproducible platform for mechanistic intervention. Consistency between the models strengthens confidence in the cellular phenotype, but validation in bovine tissues and *in vivo* models remains necessary.

Mitophagy is important for maintaining mitochondrial integrity, redox balance, cell survival, and metabolism (Pickles et al., 2018; Li et al., 2023; Zhang et al., 2024; Liu et al., 2023). In the present study, 3-MA aggravated, whereas CCCP attenuated, several BHBA-induced oxidative-stress outcomes. These findings are consistent with a protective association between mitophagy and mitochondrial quality control. However, neither compound is specific for mitophagy: 3-MA broadly affects phosphoinositide 3-kinase signaling and autophagy, whereas CCCP is a mitochondrial protonophore that induces depolarization and an integrated stress response (Koncha et al., 2021). The mitophagy intervention experiments performed in this study provide mechanistic evidence that impaired clearance of damaged mitochondria is not merely a consequence of BHBA-induced oxidative injury but also a functional contributor to its progression. Inhibition of mitophagy by 3-methyladenine (3-MA) exacerbated BHBA-induced ROS accumulation, further reduced the activities of CAT, SOD, and GSH-Px/GPX, and increased MDA and H_2_O_2_ levels. In contrast, activation of mitophagy by CCCP decreased intracellular ROS accumulation and improved the antioxidant status of cells exposed to BHBA. These findings suggest that mitophagy exerts a protective effect by facilitating the removal of damaged mitochondria and limiting excessive ROS generation. Notably, mitophagy activation enhanced the expression of genes involved in ketone body metabolism, whereas mitophagy inhibition reduced the expression of BDH1 and OXCT1. This observation further suggests a close functional link between mitochondrial quality control and ketone body utilization. Efficient ketone body metabolism depends on intact mitochondrial function (Zhu Y. X. et al., 2025).

Thus, the pharmacological experiments do not by themselves establish that the observed effects were mediated exclusively through mitophagy. Although primary bovine muscle satellite cells were used to characterize BHBA-induced mitochondrial dysfunction and alterations in mitophagy-related markers, the pharmacological intervention experiments were performed in C_2_C_12_ myoblasts due to their established utility for mitochondrial and mitophagy studies. Therefore, validation of mitophagy modulation directly in primary BMSCs remains an important direction for future investigation. Future studies should combine mitophagy-flux assays with genetic manipulation of PINK1, Parkin, BNIP3, FUNDC1, or related regulators. The study also has several broader limitations. First, all findings were obtained *in vitro* and cannot reproduce the endocrine, nutritional, inflammatory, and inter-organ complexity of ketosis in dairy cows. The results should therefore be interpreted as cellular proof of concept rather than evidence of therapeutic efficacy. Second, validation in skeletal muscle from cows with different degrees of ketosis is needed. Third, the mechanisms by which BHBA affects non-coding RNAs and mitochondrial quality-control pathways remain unresolved. Finally, NaHB was examined at only one concentration. Dose-response studies, direct mitochondrial functional assays, and *in vivo* validation will be required before the physiological and translational significance of these findings can be established. In addition, the TEM observations in this study were qualitative and intended to provide ultrastructural confirmation of mitochondrial alterations. Quantitative morphometric analyses of mitochondrial number, morphology, and autophagosome abundance were not performed, which represents a limitation of the present study. Future studies incorporating systematic TEM quantification will provide more rigorous ultrastructural validation of mitochondrial injury and mitophagy alterations.

## 5 Conclusion

In summary, our study highlights the critical interplay among BHBA accumulation, oxidative stress, mitochondrial dysfunction, and mitophagy in the pathogenesis of bovine ketosis. Elevated BHBA levels induced excessive mitochondrial ROS production, leading to oxidative stress, impaired mitochondrial function, suppressed proliferation, and enhanced apoptosis in BMSCs. Although BHBA stimulated may reflect an adaptive cellular response, including increased mitochondrial biogenesis and PGC1α expression, these adaptations were insufficient to restore mitochondrial homeostasis. Concurrently, disruption of mitochondrial dynamics and inhibition of mitophagy promoted the accumulation of dysfunctional mitochondria, further amplifying oxidative damage. Collectively, this study advances our understanding of the cellular mechanisms underlying skeletal muscle metabolic dysfunction during bovine ketosis and highlights mitophagy as a promising therapeutic target for preventing or mitigating BHBA-induced muscle injury.

## Data Availability

The original contributions presented in the study are included in the article/Supplementary Material, further inquiries can be directed to the corresponding author.
