# Supplementary material for: β-Hydroxybutyrate-induced mitochondrial dysfunction and oxidative stress in BMSCs are ameliorated by mitophagy activation
Source: Front Cell Dev Biol. 2026 Aug 27;14:1926968. doi: 10.3389/fcell.2026.1926968 (PMC13558407; doi:10.3389/fcell.2026.1926968)
Supplement: Supplementary file 1 [file Supplementaryfile1.zip › Supplementary File(s)/Supplementary File(s).docx]

**Figure S1. Expression levels of β-actin and GAPDH proteins after treatment with different concentrations of BHBA. The data presented as means ± SEM. * *P* < 0.05; ** *P* < 0.01.**

**Figure S2. The effect of different concentrations of BHBA (0 mM, 1.2 mM, 2.4mM, 4.8mM) on C_2_C_12_. (A) The picture of the EdU proliferation assay for C_2_C_12_ with BHBA treatment for 12h and the percent of EdU-positive cells (n=3). (B)** **The 450 nm absorbance of C_2_C_12_ after treatment with BHBA (n=6). (C) Relative expression level of proliferation genes of different concentrations of BHBA treatment (n=6). (D) Western blot analysis of BCL2 and BAX protein and detection of gray value (n=3). (E) Relative expression level of apoptosis genes of different concentrations of BHBA treatment (n=6). (F) JC-1 staining of mitochondrial membrane potential of different concentrations of BHBA (0 mM, 1.2 mM, 2.4mM, 4.8mM) on C_2_C_12_. (G, H) Western blot analysis and detection of gray value (n=3). (I)** **Assessment of ROS in C_2_C_12_ following treatment with different concentrations of BHBA. (J) (E) The detection of CAT, SOD, GSH-Px/GPX activity and the content of MDA and H_2_O_2_ in C_2_C_12_ after BHBA treatment (n=6). (K) Western blot analysis of SOD1, SOD2 and CAT protein. (L) Relative expression level of mitophagy genes (n=6). (M) SOD1, SOD2, CAT protein grayscale value calculation (n=3). (N) Assessment of mitochondrial ROS in C_2_C_12._ (O)** **Representative TEM micrographs of C_2_C_12_ with BHBA (0 mM, 2.4mM, 4.8mM) treatment. The red arrows represent mitochondria, the purple arrows represent autophagosomes, the blue arrows represent smooth endoplasmic reticulum expansion, RER refers to rough endoplasmic reticulum, SER refers to smooth endoplasmic reticulum, Mi refers to mitochondria, and N refers to the nucleus Scale bar=1 μm. (P-T)** **The relative expression levels of genes related to mitochondrial fusion, fission, translation, protein transport, biosynthesis, and mitochondrial complexes. The data presented as means ± SEM. * *P* < 0.05; ** *P* < 0.01.**

**Figure S3. The qPCR results of BMSCs after being treated with 0 mM, 1.2 mM, 2.4 mM, and 4.8 mM concentrations of BHBA for 12 hours.** (A) The relative expression levels of *PGC1α, FOXO1* genes related to ketone body metabolism (n=6). (B) The relative expression levels of genes related to cell proliferation, apoptosis, and oxidative stress in BMSCs treated with different concentrations BHBA (n=6). (C) The relative expression of genes related to mitochondrial autophagy, fusion, fission, and synthesis (n=6). (D, E) The relative expression levels of genes related to mitochondrial complexed, mitochondrial-encoded genes, mitochondrial protein transport genes, and genes related to mitochondrial translation (n=6).
